# Changes in arm-hand function and arm-hand skill performance in patients after stroke during and after rehabilitation

**DOI:** 10.1371/journal.pone.0179453

**Published:** 2017-06-14

**Authors:** Johan Anton Franck, Rob Johannes Elise Marie Smeets, Henk Alexander Maria Seelen

**Affiliations:** 1 Adelante Rehabilitation Centre, dept. of brain Injury Rehabilitation, Hoensbroek, the Netherlands; 2 Adelante Centre of Expertise in Rehabilitation and Audiology, Hoensbroek, the Netherlands; 3 Maastricht University, Research School CAPHRI, dept. of Rehabilitation Medicine, Maastricht, the Netherlands; University of Ottawa, CANADA

## Abstract

**Background:**

Arm-hand rehabilitation programs applied in stroke rehabilitation frequently target specific populations and thus are less applicable in heterogeneous patient populations. Besides, changes in arm-hand function (AHF) and arm-hand skill performance (AHSP) during and after a specific and well-described rehabilitation treatment are often not well evaluated.

**Method:**

This single-armed prospective cohort study featured three subgroups of stroke patients with either a severely, moderately or mildly impaired AHF. Rehabilitation treatment consisted of a Concise_Arm_and_hand_ Rehabilitation_Approach_in_Stroke (CARAS). Measurements at function and activity level were performed at admission, clinical discharge, 3, 6, 9 and 12 months after clinical discharge.

**Results:**

Eighty-nine stroke patients (M/F:63/23; mean age:57.6yr (+/-10.6); post-stroke time:29.8 days (+/-20.1)) participated. All patients improved on AHF and arm-hand capacity during and after rehabilitation, except on grip strength in the severely affected subgroup. Largest gains occurred in patients with a moderately affected AHF. As to self-perceived AHSP, on average, all subgroups improved over time. A small percentage of patients declined regarding self-perceived AHSP post-rehabilitation.

**Conclusions:**

A majority of stroke patients across the whole arm-hand impairment severity spectrum significantly improved on AHF, arm-hand capacity and self-perceived AHSP. These were maintained up to one year post-rehabilitation. Results may serve as a control condition in future studies.

## Introduction

One of the most common deficits following stroke is a persistent impairment of the arm and hand due to a hemiparesis, which has a significant impact on performance in daily life activities [1]. Recovery of arm-hand function and skills is a major rehabilitation and health care challenge. Motor rehabilitation approaches for arm-hand performance after stroke has been changing substantially over the last decades. However, an integral arm-hand skill training approach, accommodating both the heterogeneity of the patient population and its associated patterns and levels of recovery directly post-stroke seems to be absent. A large number of well-explored and well-investigated examples of training approaches in specific (sub) populations have been identified [2] like, for instance, task-oriented training [3], mental practice [4] and constraint-induced movement therapy (CIMT) [5]. In task-oriented approaches specific functional, skill-related tasks are trained. This is done preferably by using real-life objects [6], thereby teaching patients to solve specific problems related to, e.g., anticipatory motor adjustments or cognitive processing by using efficient goal-oriented movement strategies [7, 8].

Existing task-oriented arm-hand programs (e.g. [9–16]) are valuable contributions to rehabilitation practice and may offer a stable point of departure for clinicians to select the most appropriate therapy for a particular patient.

However, several aspects make it difficult for clinicians to choose the most appropriate arm-hand therapy intervention(s) for a particular patient: 1) Most studies or programs target specific populations (in particular those with some preservation of wrist and/or finger extension) and thus are less applicable for patients with a more severely affected arm-hand as seen in the heterogeneous populations of many rehabilitation centres [17]. 2) Programs are focused on either the arm or the hand alone. 3) Most of the current studies in research projects feature strictly protocolled interventions, which cannot be easily adopted in the clinicians’ daily practice. 4) The lack of information about the proportional improvement or deterioration to be expected in stroke survivors in the sub-acute phase after stroke may lead to difficulties for clinicians to make decisions about arm-hand treatment objectives and concomitant prognostics regarding arm-hand skill performance.

In order to overcome these four drawbacks a Concise Arm and hand Rehabilitation Approach in Stroke (acronym: CARAS) [18] was developed in order to guide clinicians, during their daily practice, in systematically designing a patient’s optimal arm-hand rehabilitation program. CARAS is based on four constructs: a) stratification of the patient population is based on the severity of arm–hand impairment for which the Utrechtse Arm-hand Test (UAT) is used [19], b) clear focus on the individual’s rehabilitation goals and concomitant potential rehabilitation treatment outcomes, c) principles of self-efficacy, and d) possibility to systematically incorporate (new) technology and new evidence-based training elements swiftly. CARAS has proven to be feasible in a number of stroke units of rehabilitation centres throughout the Netherlands.

In the present study, the term ‘arm–hand function’ (AHF) refers to the ICF ‘*body function and structures level’*. The term ‘arm-hand skilled performance’ (AHSP) refers to the ICF *activity level*, covering both capacity and performance [20].

The present paper focusses on two aspects.

Firstly, during rehabilitation AHF and AHSP may improve to a certain level. However, once a stroke patient has left the rehabilitation program, his arm-hand capacity and performance may deteriorate [21]. Whereas stroke patients with mild to moderate initial impairments show an almost fixed amount of recovery after stroke, ranging up to 70% [22, 23], stroke patients with a more severely affected arm-hand, i.e. absence of finger extension combined with large motor impairments, strongly lag behind this recovery percentage. Four years after stroke, 67% of stroke survivors still experience non-use or disuse of the moderately or severely affected arm–hand [24].

However, it is neither well understood at what rate such deterioration (or improvement) occurs, nor in which patient categories, i.e. patients with a certain level of arm-hand severity, this is most prominent. Answers to these questions are essential for the development of more adequate, personalised and cost-effective interventions that may augment and/or maintain arm-hand skill performance (AHSP) levels in stroke patients living in their home environment.

Secondly, the risk of losing the opportunity to clearly define ‘therapy-as-usual’ (TAU) is becoming a problem in AHSP research in stroke patients. In the myriad of studies evaluating newly developed training protocols aimed at improving AHF and/or AHSP, each of these new training approaches is contrasted to some kind of TAU, the latter of which may vary widely between clinics and institutes. Even worse, often TAU is not clearly defined at all.

As the implementation of many of the tested experimental treatments progresses, the concept of ‘therapy-as-usual’ inevitably will be lost.

The aim of the present study was to evaluate the course AHF and AHSP take in a broad range of sub-acute stroke patients during and after rehabilitation involving a therapy-as-usual (i.e. CARAS) [18].

Three subgroups, i.e. a subgroup of patients with a severely affected arm-hand, a subgroup of patients with a moderately affected arm-hand and a subgroup of patients with a mildly affected arm-hand, were formed.

The research questions were:

1. To what extent do arm-hand function and arm-hand skill performance in stroke patients change during and after their rehabilitation involving therapy-as-usual?
2. To what extent does the rate of improvement or deterioration (over time) of arm-hand function and arm-hand skill performance differ between three subgroups of stroke patients, i.e. patients with either a severely, moderately or mildly affected functional arm-hand, during and after their rehabilitation involving CARAS?

## Methods

### Design

This study is a single-armed prospective cohort study conducted between February 2011 and May 2015. Stroke patients who experienced AHF loss and (concomitantly) AHSP loss were assessed during and up till 12 months after their protocolled rehabilitation treatment.

This investigation has been conducted according to the principles expressed in the Declaration of Helsinki. This project was approved by the Medical Ethics Committee of Maastricht University Medical Centre in the Netherlands (dossier number NL35681.068.11).

Written informed consent was obtained from all participants prior to the start of their participation in this study.

### Population

The study population consisted of a broad range of sub-acute stroke patients admitted to an inpatient stroke ward of the Adelante Rehabilitation Centre. Identification of potential participants was done by the rehabilitation specialists of the stroke unit, based on the inclusion and exclusion criteria as mentioned below. Inclusion criteria were kept to a minimum: age ≥18 years; clinically diagnosed with central paresis of the arm/hand at entry in the study; ability to control sitting posture; a fair cognitive level, i.e. being able to understand the questionnaires and measurement instructions. Exclusion criteria were: additional complaints that may interfere with the execution of the measurements; no informed consent.

In this study the primary outcome measure to assess arm-hand use on the hemiparetic side in stroke patients was the ARAT [25].Given a mean difference of at least 10% between baseline values and post-intervention values, an expected standard deviation of the difference of 15%, a two-sided test in a repeated-measures design, an alpha of 0.05, a power of 0.90, and a loss to follow-up of 10%, at least 29 participants per group needed to be included in the study. For three groups 87 participants were needed.

### Procedures

#### Therapy-as-usual

All participants followed the Concise Arm and hand Rehabilitation Approach in Stroke (CARAS) [18] as provided by the medical and paramedical staff of Adelante rehabilitation centre. CARAS spans the full range of arm-hand impairments considered for this patient group. This approach consists of a well-described program offering stepwise, transparent and comprehensible procedures, tailored to specific needs of the individual patient.

Based on the UAT score (0–7) [19], patients were allocated to one of the three subgroups (i.e. subgroup 1 = severely affected, subgroup 2 = moderately affected and subgroup 3 = mildly affected) and were allocated to one of three training programs within CARAS. Subgroup 1 followed program 1 which targets stroke survivors with an UAT score of 0 to 1, and is titled ‘*taking care and prevention’*. It is designed for stroke survivors who are not able to use their affected arm and hand for skill performance in daily life situations (non-functional arm-hand). This program contains different topics aimed at getting and keeping the affected shoulder and arm-hand in an optimal condition and learning strategies on what to do when discomfort arises. Subgroup 2 (UAT score 2–3) was admitted to program 2 and subgroup 3 (UAT score 4–7) followed program 3. Both programs are *high intensity*, *task-oriented arm-hand performance training* programs in which patients learn to integrate their affected arm and hand in daily occupations, thus optimizing their overall functional abilities in daily situations. Patients in subgroup 2 will work on becoming more able to use their affected arm and hand for passive and active stabilisation tasks, like fixating bread while making a sandwich. Patients from subgroup 3 are already able to use their affected arm and hand instantaneously in daily situations. They will work more towards complex (bi-)manual activities. Patients in program 1 spend 4.5 hours on training spread across each week. Patients following program 2 or 3 receive an intensive exercise training of 6 hours spread across each week. A more detailed description of the therapy content and basic assumptions of CARAS have been described by Franck and co-workers [18].

After baseline measurements, patients enrolled in one of the three programs and started training for six consecutive weeks. After six weeks the patient left the program and entered the second assessment. Progression made, was expressed in terms of functional goals reached, based on performance and capacity levels exceeding certain minimal clinically important thresholds as captured by outcome measurements. Depending on these results it was possible for the patient to choose for a second (and final) six weeks period of training, which was then also evaluated.

#### Outcome measures

The following measurements, covering both the ‘function’ and ‘activities’ domain of the International Classification of Function, Disability and Health (ICF) (World_Health_Organization, 2001), were performed.

At function level, the Fugl-Meyer Motor Assessment (FM) and dynamometry (gauging grip strength) were used. The upper extremity section of the FM is a reliable and valid test for the assessment of arm-hand function in stroke patients at function level [26, 27]. Its score ranges from 0 to 66. The minimal clinical important difference (MCID) for the FM upper extremity section is 9 points (for both the affected dominant and affected non-dominant arm-hand) [28].

Grip strength of the hand was measured using the JAMAR hand dynamometer [29]. Grip strength (in N) was measured three times and the mean score was used. The MCID for grip strength were 50 N (affected dominant hand) and 62 N (affected non-dominant hand) [30].

At activity level, encompassing both capacity and perceived performance, the Action Research Arm test (ARAT) and ABILHAND were used. The ARAT has been proven to be reliable, valid and sensitive to change in measuring upper limb capacity at activity level in patients with stroke [31–34]. It consists of four subtests comprising 16 grasp movements and three reaching movements to be performed by the patient. Items are scored on a 4-point scale, its sum score ranging from 0 to 57. The MCID of the ARAT were 12 points (affected dominant arm-hand) and 17 points (affected non-dominant arm-hand) [30].

The ABILHAND is a Rasch-evaluated test to assess the manual ability in terms of the difficulty perceived by patients with hand impairments in their daily life [35]. It focuses on 23 representative unimanual or bimanual activities [36, 37]. The test is administered as a semi-structured interview, using a 3-level ordinal rating scale: impossible (0), difficult (1), and easy (2) to perform. The ABILHAND is valid, responsive and clinically useful [35, 36]. The MCID of the ABILHAND is within a range of 0.26 to 0.35 [38].

Finally, one single question was used every two weeks to gauge the occurrence of any major event (for example ‘flu’, ‘falls’, etc.) that might have affected the use of the arms or hands of the patient over the past two weeks. (“Has there been any major problem during the last 2 weeks preventing you from using one or both hands? (yes/no). If so, please indicate (from a short list) which event(s).”). This question was merely used as an indicator in case of any unexplainable data shift in a patient’s time series occurring. This indicator was not used in the statistical analyses.

In clinical practice and in randomized clinical trials an improvement of 10% or more on the primary outcome measure is often considered being clinically relevant (e.g. Kwakkel et al [39] [40]). As to deterioration on a primary outcome measure, no clear guidelines are available. In our study we therefore decided to use a conservative threshold of 5% in identifying any deterioration, thus making sure that even a small reduction in the outcome would be considered being a deterioration.

#### Measurement dates

**Fig 1:**
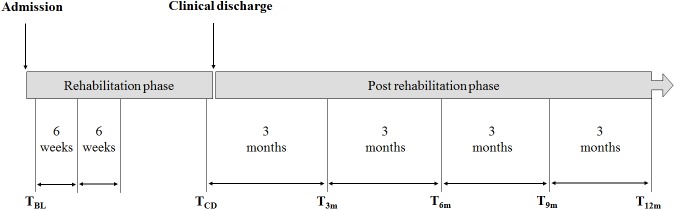
Overview of measurement timing. T = time; BL = baseline; CD = clinical discharge; m = month.

As soon as possible after admission to the rehabilitation centre the aforementioned measurements were performed (baseline measurement (T_BL_)). Furthermore, at five additional points in time, interspaced by three months, measurements were performed, starting one week prior to discharge from the clinical and outpatient rehabilitation program (T_CD_), followed by T_3m_ (= T_CD_ + 3 months) through T_12m_ (= T_CD_ + 12 months). In Fig 1 an overview of the measurement dates is given.

#### Data processing and statistical analysis

For data representation, boxplots were used. As to the methodological handling of missing values in this study, four decision rules, formulated prior to the start of the study, were applied (consecutively).

1. When the baseline value was missing, its value was estimated using the mean baseline value of the (sub-)group.
2. When the T_12m_ value was missing, the ‘last observation carried forward’ procedure [41] was used.
3. When 1 or 2 (temporally adjacent) value(s), not being the baseline value or the T_12m_ value, were missing, these missing value(s) were estimated by linear interpolation using the two valid adjacent values in the time series.
4. In case of 3 or more missing values, the whole case was discarded.

The MCID threshold values, as used in this study, were corrected for hand dominance. Per subgroup, the number of patients whose outcome scores exceeded these MCID thresholds were ascertained. These numbers were then converted into a percentage of the total number of persons within a subgroup.

In order to assess whether data were normally distributed or not, multiple Shapiro-Wilk tests were performed. As, eventually, nearly all data turned out to be non-normally distributed, data were statistically analysed using non-parametric statistics. Alpha was set at 0.05. The ‘intention-to-treat’ principle was used, i.e. patients were analysed according to the program in which they originally started. Friedman two-way analysis of variance by ranks was used for the data of each subgroup to determine whether patients improved over time, i.e. between all measurement dates from T_BL_ up till T_12m_ regarding the FM, grip strength, ARAT, and ABILHAND. Subsequent multiple comparisons between T_BL_ and T_CD_ data (to ascertain any changes in AHF and AHSP during the rehabilitation phase) as well as between T_CD_ and T_12M_ data (representing changes the one year follow-up), included Wilcoxon signed ranks tests. A Bonferroni approach was used to control for spurious false positive findings.

For ascertaining any possible differences between subgroups as to the rate of improvement in arm-hand performance during rehabilitation and during the 12 months follow-up, Kruskal-Wallis tests were applied. Here, multiple comparisons included Mann-Whitney U-tests in a Bonferroni approach. Data were analysed using SPSS software (version 23.0, IBM Corp., New York).

## Results

### Error analysis

In total 89 patients entered the study. Three patients dropped out due to a recurring stroke during the study. Two patients prematurely left the study after the T_CD_ measurement because of personal reasons. Six patient cases were discarded following the procedures we used regarding missing values (rule number 4), i.e. when data from three or more measurement dates were missing. No significant differences in patient characteristics existed between the patients who dropped out and the remaining patients.

Shapiro-Wilk tests revealed that the majority of data were not normally distributed. Therefore, data were analyzed using non-parametric statistics as described earlier.

### Patient characteristics

The characteristics of the 89 patients included in the study are shown in Table 1.

**Table 1:** Patient characteristics upon inclusion in the study.

Characteristics	Whole group	Subgroups		
		1	2	3
**Total number (n)**	89	28	28	33
**Age (y), mean (sd)**	57.6 (10.6)	56.2 (11.0)	57.9 (12.5)	58.5 (8.5)
**Gender (n, %)**				
Male	63 (70.8%)	15 (53.6%)	24 (85.7%)	24 (72.7%)
Female	26 (29.2%)	13 (46.4%)	4 (14.3%)	9 (27.3%)
**Side of lesion (n, %)**				
Left	53 (59.6%)	13 (46.4%)	18 (64.3%)	22 (66.7%)
Right	36 (40.4%)	15 (53.6%)	10 (35.7%)	11 (33.3%)
**Stroke Type (n, %)**				
Haemorrhagic	17 (19.1%)	5 (17.9%)	5 (17.9%)	7 (21.2%)
Ischemic	72 (80.9%)	23 (82.1%)	23 (82.1%)	26 (78.8%)
**Lesion site as diagnosed (n)**				
Basal ganglia	7	1	2	4
Brainstem	2			2
Capsula interna	1			1
Cerebellum	2			2
Frontal area	2		1	1
Frontoparietal area	1	1		
Frontotemporal area	2	1	1	
Parietal area	1			1
Parietotemporal area	1	1		
Posterior area	1			1
Temporal area	1			1
Temporal area & thalamus	1			1
Thalamus	4	1	1	2
Pontine	1			1
Hemisphere	54	22	20	12
Lacunar	5		3	2
Medulla oblong. & cerebellum	1			1
Nucleus caudatus	2	1		1
**Time post stroke (days), mean (sd)**	29.8 (20.1)	40 (27.5)	27 (14.5)	23.4 (12.6)
**Affected hand (n)**				
Dominant	50	11	17	22
Non-dominant	39	17	11	11

n = number

sd = standard deviation.

### Improvement over time within each subgroup

#### Fugl-Meyer assessment

**Fig 2:**
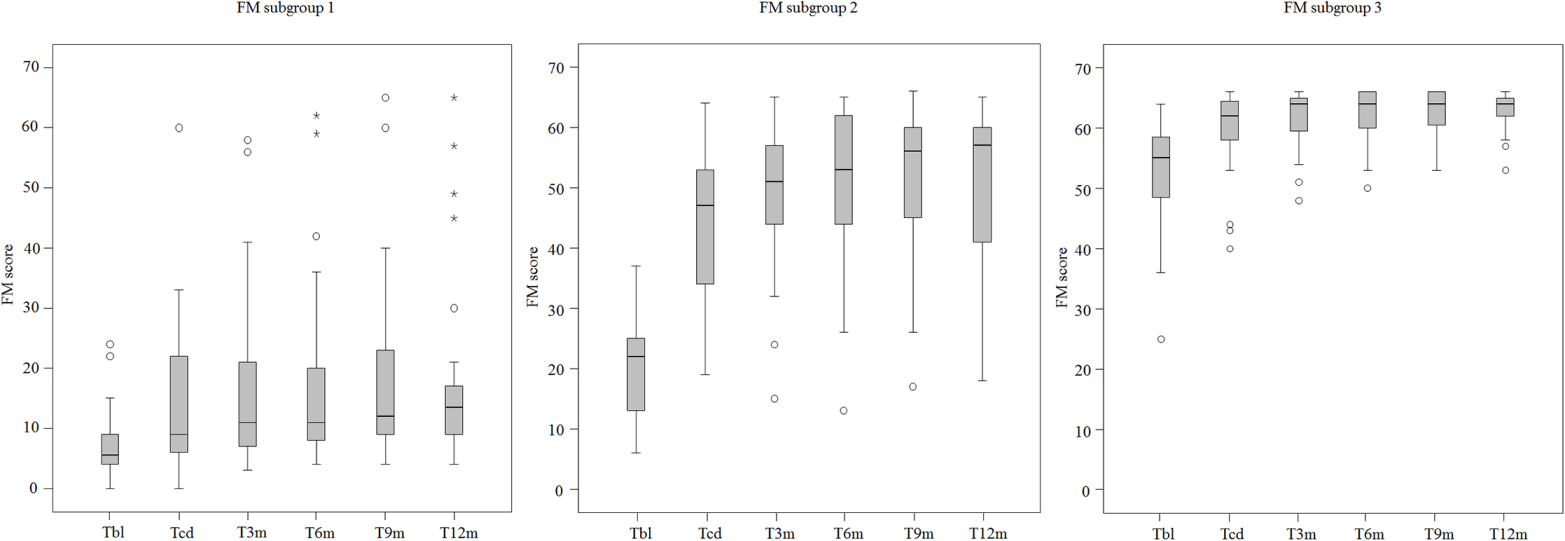
Boxplots of the FM data per measurement moment for each of the three groups. FM = Fugl Meyer Motor Assessment; T_BL_ = Baseline; T_CD_ = clinical discharge; T_3m_ = (= T_CD_ + 3 months); T_6m_ (= T_CD_ + 6 months); T_9m_ (= T_CD_ + 9 months);T_12m_ (= T_CD_ + 12 months). Circle = outlier value; Asterisk = far out value.

In Fig 2A–2C the boxplots of the FM data per measurement moment for each of the three groups are presented.

Overall, patients of the three subgroups improved over time between T_BL_ and T_12m_ on the FM (p< = 0.000). Multiple comparison analyses revealed that in subgroup 1 the FM improved between T_BL_ and T_CD_ (p = 0.003) and between T_CD_ and T_12m_ (p = 0.009). In subgroup 2 significant improvements between T_BL_ and T_CD_ (p< = 0.000) and between T_CD_ and T_12m_ (p = 0.004) were found. Similarly, in subgroup 3 improvements between T_BL_ and T_CD_ (p< = 0.000) and T_CD_ and T_12m_ (p = 0.002) were found regarding the FM scores.

#### Grip strength

**Fig 3:**
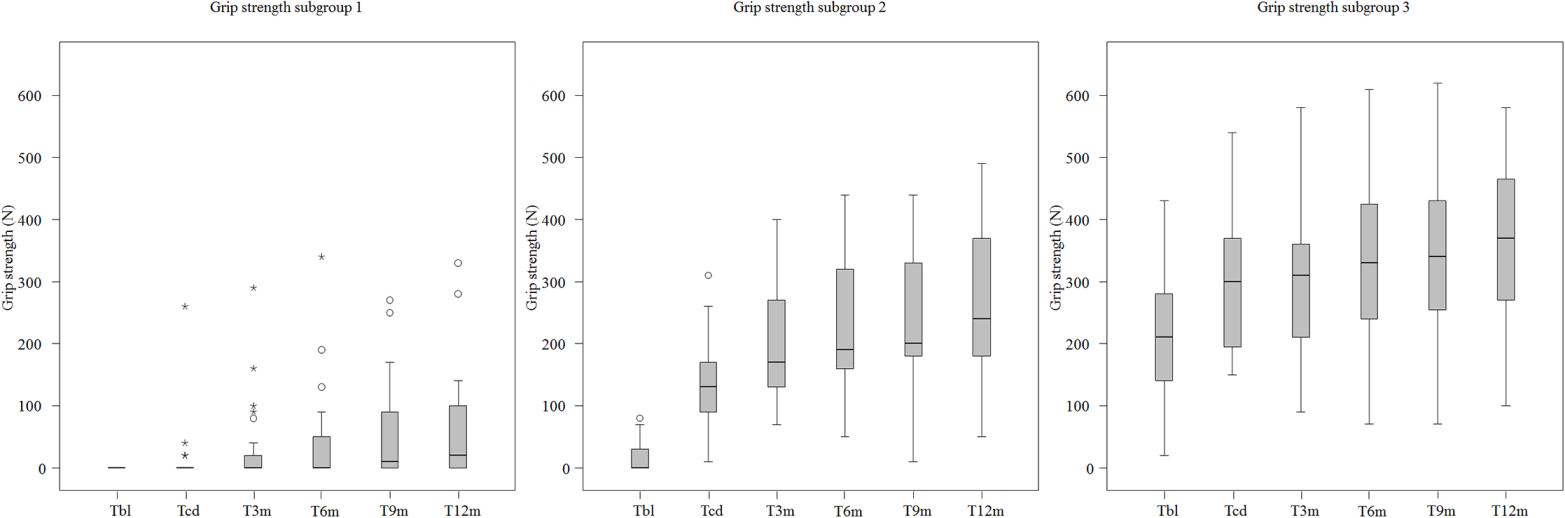
Boxplots of the grip strength data per measurement moment for each of the three groups. T_BL_ = Baseline; T_CD_ = Clinical Discharge; T_3m_ = (= T_CD_ + 3 months); T_6m_ (= T_CD_ + 6 months); T_9m_ (= T_CD_ + 9 months);T_12m_ (= T_CD_ + 12 months). Circles = outlier value; Asterisk = far out value.

In Fig 3A–3C the boxplots of the grip strength data per measurement moment for each of the three groups are presented.

In general, patients of the three subgroups improved on grip strength between T_BL_ and T_12m_ (p< = 0.000). In subgroup 1 no significant changes between T_BL_ and T_CD_ (p = 0.066) were found, in contrast to the follow-up period, i.e. between T_CD_ and T_12m_, in which substantial improvements were found (p = 0.001). Subgroup 2 and subgroup 3 showed a significant improvement as to grip strength between T_BL_ and T_CD_ (p< = 0.000), and, similarly, in the follow-up period between T_CD_ and T_12m_ (p< = 0.000).

#### Action research arm test

**Fig 4:**
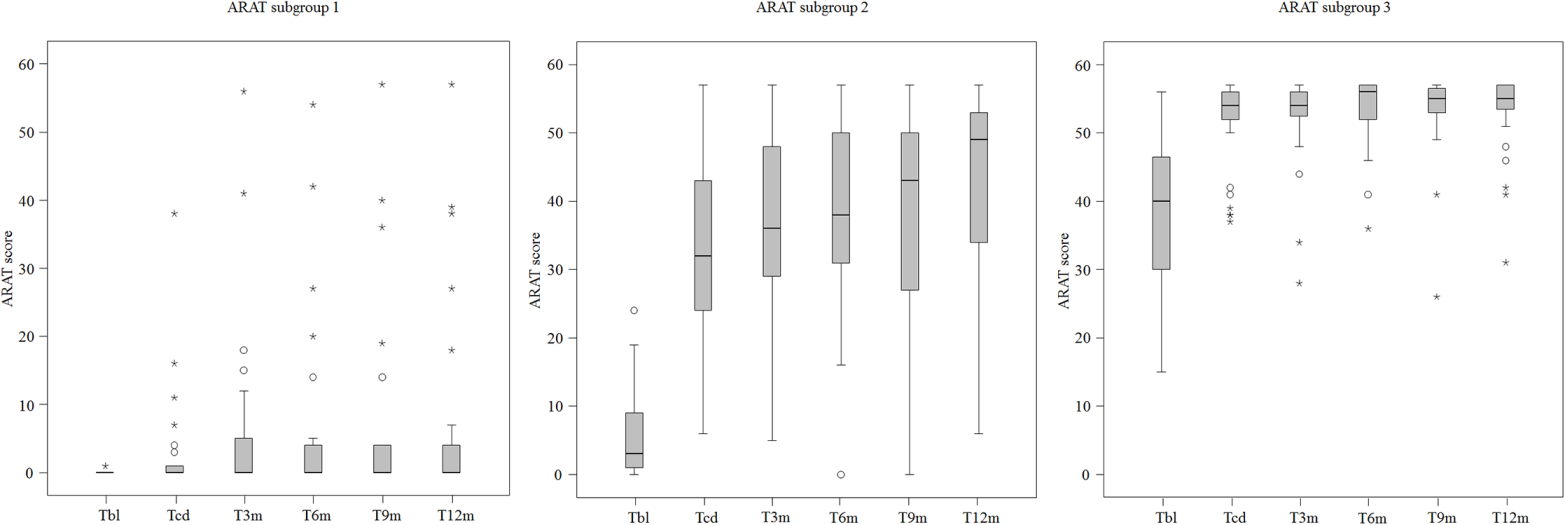
Boxplots of the ARAT data per measurement moment for each of the three groups. ARAT = Action Research Arm Test; T_BL_ = Baseline; T_CD_ = Clinical Discharge; T_3m_ = (= T_CD_ + 3 months); T_6m_ (= T_CD_ + 6 months); T_9m_ (= T_CD_ + 9 months);T_12m_ (= T_CD_ + 12 months). Circles = outlier value; Asterisk = far out value.

In Fig 4A–4C the boxplots of the ARAT data per measurement moment for each of the three groups are presented.

Overall, patients of the three subgroups improved on the ARAT over time between T_BL_ and T_12m_ (p< = 0.000). Subgroup 1 progressed on the ARAT between T_BL_ and T_CD_ (p = 0.018) and in the follow-up period between T_CD_ and T_12m_ (p = 0.007). In subgroup 2 progression was found between T_BL_ and T_CD_ (P< = 0.000), and between T_CD_ and T_12m_ (p = 0.001). Subgroup 3 improved on the ARAT between T_BL_ and T_CD_ (p< = 0.000). However, in the latter group, no significant changes on the ARAT were found between T_CD_ and T_12m_ (p = 0.071).

#### ABILHAND

**Fig 5:**
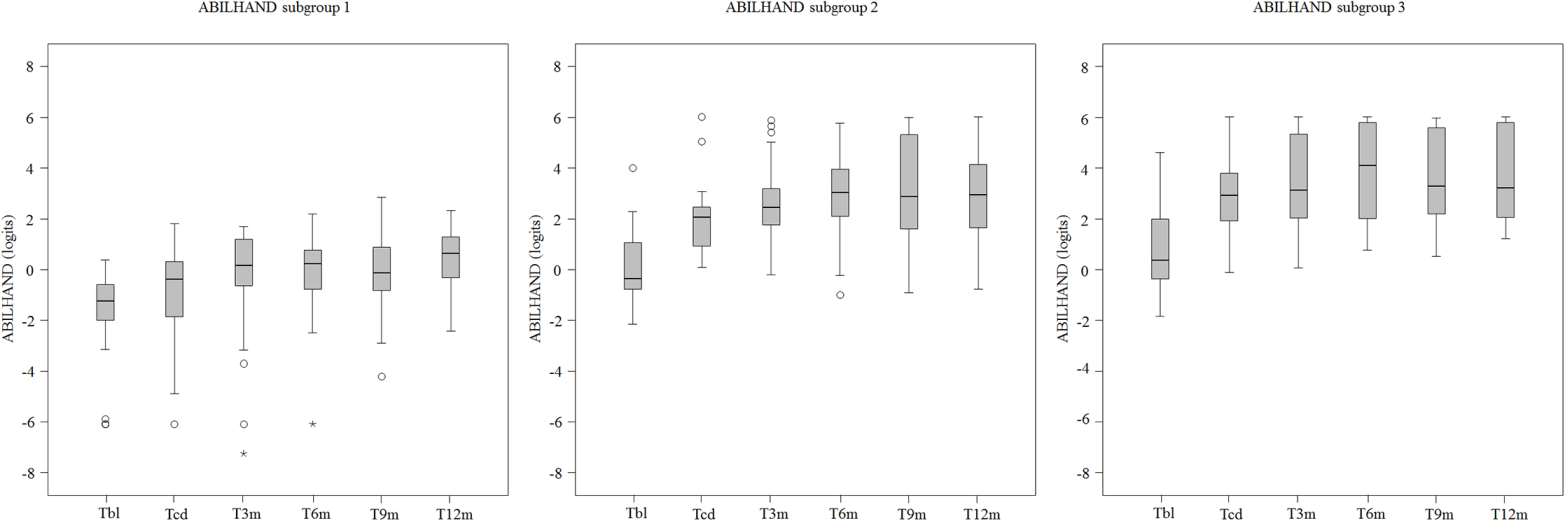
Boxplots of the ABILHAND results per measurement moment for each of the three groups. T_BL_ = Baseline; T_CD_ = Clinical Discharge; T_3m_ = (= T_CD_ + 3 months); T_6m_ (= T_CD_ + 6 months); T_9m_ (= T_CD_ + 9 months);T_12m_ (= T_CD_ + 12 months). Circles = outlier value; Asterisk = far out value.

In Fig 5A–5C the boxplots of the ABILHAND data per measurement moment for each of the three groups are presented.

Generally speaking, patients of subgroup 1, 2 and 3 improved on the ABILHAND over time (p< = 0.000). Subgroup 1 significantly progressed on the ABILHAND between T_BL_ and T_CD_ (p = 0.014), and between T_CD_ and T_12m_ (p< = 0.000). In subgroup 2 improvements on the ABILHAND were found between T_BL_ and T_CD_ (p< = 0.000), but not between T_CD_ and T_12m_ (p = 0.044). Subgroup 3 improved on the ABILHAND between T_BL_ and T_CD_ (p< = 0.000), but not between T_CD_ and T_12m_ (p = 0.040).

### Deterioration of arm-hand function, capacity and performance post-rehabilitation

In Table 2 the number and percentage of patients per subgroup whose arm-hand function, arm-hand capacity and/or performance either deteriorated (more than 5% relative to clinical discharge performance), or remained equal or improved during the *post-rehabilitation* phase, are presented. Deterioration of arm-hand function, as measured with the FM, occurred in 19.2% of the persons with a severely affected arm-hand. In persons with a moderately or mildly impaired arm-hand this occurred in 19.0% and 3.2%, respectively. Deterioration of grip strength occurred in 0% of the severely affected subgroup patients, in 9.5% of the moderately impaired subgroup and in 3.2% of the mildly affected subgroup patients. Arm-hand capacity, as measured with ARAT, showed a deterioration of 0% in the severely affected group, 9.5% in the moderately affected subgroup and 3.2% in the mildly affected subgroup. Deterioration of perceived performance, as measured with the ABILHAND, occurred in 56.0% of persons with a severely affected arm-hand, in 23.8% and 29.0% of the moderately and mildly impaired persons, respectively.

**Table 2:** Number (and %) of patients per subgroup whose arm-hand capacity and/or performance either deteriorated (more than 5% relative to clinical discharge performance), or remained equal or improved during the *post-rehabilitation* phase.

Test	Worse or Equal / better		Subgroups	
		1	2	3
		(n = 26)	(n = 21)	(n = 31)
**FM**				
	worse	5 (19.2%)	4 (19.0%)	1 (3.2%)
	equal / better	21 (80.8%)	17 (81.0%)	30 (96.8%)
**Grip strength**				
	worse	0 (0.0%)	2 (9.5%)	1 (3.2%)
	equal / better	26 (100%)	19 (90.5%)	30 (96.8%)
**ARAT**				
	worse	0 (0.0%)	2 (9.5%)	1 (3.2%)
	equal / better	26 (100%)	19 (90.5%)	30 (96.8%)
**ABILHAND**				
	worse	14 (53.8%)	5 (23.8%)	9 (29.0%)
	equal / better	*11 (46.2%)	16 (76.2%)	22 (71.0%)

FM = Fugl Meyer test; ARAT = Action Research Arm Test; Asterisk = one data point in 1 patient (P10) regarding the ABILHAND is missing for Tcd.

### Differences in rate of improvement over time between subgroups

In Table 3 the rates of improvement over time of subgroups regarding the FM, grip strength, ARAT and ABILHAND are presented.

**Table 3:** Rate of improvement over time (mean, sd, median) of patients regarding FM, ARAT, grip strength and ABILHAND.

Test	Time phase				Subgroups						P-value			
		1 (n = 26)			2 (n = 21)			3 (n = 31)			overall	Gr1—Gr2	Gr1—Gr3	Gr2—Gr3
**FM**		Mean	sd	Median	Mean	sd	Median	Mean	sd	Median				
	T_bl_—T_cd_	6.7	13.3	2.0	22.5	12.5	24.0	7.1	7.3	7.0	0.000	0.000	0.064	0.000
	T_cd_—T_12m_	4.7	9.7	2.0	8.1	11.4	7.0	3.2	5.9	1.0	0.177	-	-	-
**Grip Strength**														
	T_bl_—T_cd_	1.3	5.1	0.0	11.9	7.1	12.0	6.9	8.0	7.0	0.000	0.000	0.000	0.029
	T_cd_—T_12m_	4.6	6.7	2.0	11.4	8.6	10.0	7.6	7.8	5.0	0.009	0.005	0.050	0.078
**ARAT**														
	T_bl_—T_cd_	3.0	8.1	0.0	27.7	12.1	28	13.1	11.5	11.0	0.000	0.000	0.000	0.000
	T_cd_—T_12m_	4.6	12.6	0.0	7.6	8.0	6.0	1.3	3.7	1.0	0.004	0.008	0.684	0.001
**ABILHAND**														
	T_bl_—T_cd_	0.94	2.09	0.85	1.97	1.63	2.49	2.38	1.95	2.13	0.056	-	-	-
	T_cd_—T_12m_	1.23	1.52	0.61	0.82	1.56	0.66	0.68	1.57	0.90	0.567	-	-	-

FM = Fugl Meyer test; ARAT = Action Research Arm Test; T.. = time; bl = baseline; cd = clinical discharge; 12m = 12 months follow-up time; Gr = group; Grey cells = non-significance values;— = not tested due to non-significant overall test outcome; sd = standard deviation.

#### Fugl-Meyer assessment

Overall, the rate of improvement on the FM between T_BL_ and T_CD_ differed between groups (p< = 0.000). Furthermore, FM rate of improvement between T_BL_ and T_CD_ differed between group 1 and 2 (p< = 0.000), between group 2 and 3 (p< = 0.000) but not between group 1 and 3 (p = 0.064).

In general, no significant differences between groups regarding the rate at which FM scores changed between T_CD_ and T_12m_ were found (p = 0.177).

#### Grip strength

Overall, changes in grip strength between T_BL_ and T_CD_ differed between the three subgroups (p< = 0.000). More specifically, the rate of grip strength improvement differed significantly between group 1 and 2 (p< = 0.000), group 2 and 3 (p = 0.029) and between group 1 and 3 (p< = 0.000).

With respect to the T_CD_ and T_12m_ measurement period, overall, differences as to the rate at which grip strength improved were found between the groups (p = 0.009). Multiple comparisons revealed a significant difference in the rate of improvement between group 1 and 2 (p = 0.005). However, no significant differences in the rate of improvement on grip strength were found between group 1 and 3 (p = 0.050) or group 2 and 3 (p = 0.078).

#### Action research arm test

Overall, the rate at which the ARAT improved between T_BL_ and T_CD_ differed among the three groups (p< = 0.000). Multiple comparisons revealed these differences to be present between all (three) combinations of subgroups (p< = 0.000).

Also, overall significant differences between the rate of improvement on the ARAT were found between the three subgroups (p = 0.004) regarding T_CD_ and T_12m_. Further analysis showed statistically significant differences in improvement on the ARAT between group 1 and 2 (p = 0.008), group 2 and 3 (p = 0.001), but not between group 1 and 3 (p = 0.684).

#### ABILHAND

Overall, differences in the rate at which patients improved on the ABILHAND between T_BL_ and T_CD_ were not statistically significant between groups (p = 0.056). Regarding the T_CD_ and T_12m_ follow-up phase, patients from the three subgroups also did not significantly differ as to their rate of improvement on the ABILHAND over time (p = 0.567).

### Percentages of patients whose outcome scores exceeded the MCID thresholds

In **Table** 4, for the FM, grip strength, ARAT and ABILHAND, the number of patients whose outcome scores exceeded the MCID thresholds, expressed as the percentage of the total number of persons within each subgroup, are presented.

**Table 4:** Number of patients whose outcome scores exceeded the MCID thresholds, expressed as the percentage of the total number of persons within a subgroup, for FM, grip strength, ARAT and ABILHAND.

Test	Time phase		Subgroups	
		1	2	3
**FM**	T_bl_—T_cd_	28%	86%	84%
	T_cd_—T_12m_	16%	38%	16%
**Grip strength**	T_bl_—T_cd_	4%	81%	61%
	T_cd_—T_12m_	38%	76%	68%
**ARAT**	T_bl_—T_cd_	4%	81%	57%
	T_cd_—T_12m_	8%	24%	3%
**ABILHAND**	T_bl_—T_cd_	66%	81%	97%
	T_cd_—T_12m_	66%	62%	65%

FM = Fugl Meyer test; ARAT = Action Research Arm Test; T.. = time; bl = baseline; cd = clinical discharge; 12m = 12 months follow-up time

## Discussion

The aim of the present study was to investigate a) to what extent arm-hand function (AHF) and arm-hand skill performance (AHSP) in subacute stroke patients change during and after their rehabilitation involving a well-described ‘therapy-as-usual’, and b) to what extent the rate of improvement or deterioration (over time) of AHF and AHSP differs between three subgroups of stroke patients. These patients are grouped according to their initial level of arm-hand impairment, i.e. severe (UAT 0–1), moderate (UAT 2–3) and mild (UAT 4–7) impairment. This study has been performed in a large stroke patient group typically seen in daily medical rehabilitation practice, i.e. only few inclusion and exclusion criteria were used, covering a broad spectrum of arm-hand problem severity levels, thus enhancing clinical generalisability of our results.

### Within-group results

With respect to the first research question: On average, patients in all three subgroups, i.e. suffering from either a severely, moderately or mildly impaired arm-hand, improved over time regarding their arm-hand function and capacity as measured with the FM, JAMAR and ARAT. More specifically, both during and after the rehabilitation phase, improvement on arm-hand capacity was observed in all three subgroups, except for the grip strength in the severely impaired group, which remained low during the rehabilitation phase, but did improve slightly during the 12 months post-rehabilitation. Given the late onset of the latter, it is very unlikely that this improvement of grip strength was caused by spontaneous recovery, whereas an increase in using the affected hand in assistance during daily living activities might explain this finding. Alternatively, as seen in many cases, the slight improvement in grip strength in the low functional group may have been caused by upcoming associated reactions, i.e. spasticity, in a later phase after stroke. This slight improvement in grip strength can be used by patients in their daily hand performance, although in most cases the functional benefits are minimal due to the negative side effects of associated reactions limiting the working range of the arm and hand.

During the rehabilitation phase, patients with an initially mildly or moderately affected arm-hand (subgroup 2 and 3) improved considerably as to arm-hand capacity and arm-hand function. Both groups were able to maintain these high levels during the post-rehabilitation phase. It should, however, be noted that, for both the FM and ARAT, patient scores in the mildly impaired subgroup related to the post-rehabilitation phase converged towards the maximum of the scale, thus constituting ceiling effects. Whether or not some of these patients may have further improved their arm-hand function and capacity beyond the scales’ ranges during the post-rehabilitation phase is unclear, especially given the fact that grip strength and perceived arm-hand performance did significantly increase in these patients during this phase.

Remarkably, seven patients who were diagnosed with an UAT score of 0–1 at baseline improved considerably as to their arm-hand capacity during and after the rehabilitation phase. These patients appear to represent a clinically meaningful subpopulation, distinctly different to the majority of patients across the low functional subgroup. Also the significant progressions observed at AHF and AHSP level between T_BL_ and T_CD_ within the moderately impaired group (group 2) leads to further questions concerning possible compensation and recovery mechanisms occurring at brain level in the sub-acute phase post-stroke. Improvements made regarding voluntary movement during and after the rehabilitation phase may be related to a substantial recovery of the corticospinal tract [42]. FM outcome (arm-hand part) is said to be associated with cortico-spinal tract integrity and may be used in predicting recovery from motor impairment after stroke [43, 44]. The progressions measured with the FM between T_BL_ and T_CD_ within this moderately impaired group suggests that a certain degree of recovery of the cortico-spinal tract may have taken place. They underpin the observation that especially persons who are classified as ‘moderately impaired’ may go through a considerable recovery process regarding their affected arm and hand.This means that patients who lack any dexterity, i.e. finger extension at the start of program 2, still have an ability to generate and maintain significant progressions in AHF and AHSP. In terms of optimization of personalized arm-hand treatment and outcome, assessment should be focused on early detection of these patients among the population of patients with an initially low-functioning arm-hand.

In order to identify persons who initially show no recovery of hand movements, transcranial magnetic stimulation (TMS) [45] and diffusion tensor imaging (DTI) [46] are upcoming techniques to accurately predict arm-hand recovery. These techniques could be especially useful in the early phase of the rehabilitation process in assisting in deciding on rehabilitation goals and concomitant therapies.

As to self-perceived AHSP measured with the ABILHAND, as gauged using the ‘5% threshold’ criterion, on average, all three groups improved over time. Whereas the severely impaired subgroup improved during both the rehabilitation phase and the post-rehabilitation phase, patients with an initially moderately or mildly impaired arm-hand function improved only significantly during the rehabilitation phase. During the post rehabilitation phase they were, again on average, able to maintain this self-perceived ability level, but did not increase any further. However, a more in-depth analysis of the post-rehabilitation data revealed that in 5 to 14% of all cases self-perceived AHSP deteriorated between clinical discharge and one-year follow-up, especially in the low functional group. However, when using the minimally clinically important difference (MCID) criterion in evaluating the ABILHAND data, a somewhat higher percentage of patients from subgroup 1 indicate having improved post-clinically. The self-perceived deterioration of AHSP by these patients is not in line with the results from more objectively quantified performance measures on capacity (ARAT) and function level (grip strength), which, for the larger part, remained at the same level or improved during the post-rehabilitation phase. One should, however, keep in mind that both ARAT and grip strength at clinical discharge were still low in a number of patients, especially in the (initially) low functioning group, and thus may have constituted so-called ‘floor effects’.

### Between-group results

With respect to the second research question: As to the potential differences in the rate of improvement between subgroups, there seems to be a non-linear, inverse U-shaped relation between the rate of improvement at the level of AHF and AHSP on the one hand, and the severity of the loss of arm-hand function directly post-stroke. The largest gains as to AHF and AHSP, both during and after the rehabilitation phase, are seen in patients with an initially moderately affected arm-hand due to a stroke (UAT score 2–3), which is consistent with studies reported by Winters [23], Prabhakaran [22], Duncan [47] and Mirbagheri [48]. However, one should keep in mind that the majority of the patients with a moderately impaired arm-hand (in contrast to persons with a severely or mild impaired arm-hand) received a second six weeks period of training.

Furthermore, in contrast to patients with a moderately affected arm-hand, patients with an initially severely (UAT score 0–1) or mildly (UAT score 4–7) affected arm-hand showed a limited rate of improvement regarding the FM and ARAT. In the latter group, however, ceiling effects in these measures as mentioned previously, might be responsible for this finding.

In contrast to the progressions made at function level and capacity level, no significant differences between the rate of improvement at self-perceived performance level between subgroups were found, neither during nor after the rehabilitation phase. Nevertheless, what becomes clear from our data is that, across all subgroups, changes in self-perceived performance regarding arm-hand skills do not correlate well with changes in a patient’s arm-hand skill capacity. Regarding the patients whose self-perceived performance decreases, the specific question arises what may have caused this deterioration in the post-rehabilitation phase. The answer to this question may be two-fold. First, one could argue that some patients’ frame of reference regarding their perception and cognitions as to their daily skill performance may (negatively) change over time. This seems plausible as patients, once they are back in their own home environment, face daily reality and daily routine and may have difficulties to cope with this. Once in post-rehabilitation, i.e. chronic phase, patients may undergo a growing awareness as to their (in)ability in arm-hand performance. However, this topic should be investigated in-depth before a more definitive explanation can be provided. Finally, the ABILHAND, gauges a patient’s performance on 23 fixed (bi)manual skills, not all of which each patient will perform in his daily routines. However, in the rehabilitation phase the patient seems to perceive his own performance more positively, in contrast to the post-rehabilitation phase in which he experiences more difficulties in the daily life circumstances. This will inevitably yield low sub-scores on some skills being encoded as having become more difficult. A possible solution to this problem may be to further personalize the list of skills to better fit the patient’s changing needs and skill ambitions across time, which currently is beyond the scope of the ABILHAND.

### Strength and limitations of this study

Most evidence-based therapies that have been shown to be effective for arm-hand performance in the post-stroke phase, are based on the testing of a single experimental intervention relative to various shapes of ‘therapy-as-usual’ in a group of preselected patients with at least some residual arm-hand function, predominantly focussing on persons with a mildly affected arm and hand. However, research studying the performance of patients with an unfavourable prognosis and/or a non-functional hand (i.e. UAT score 0–1 and 2–3) are scarce in literature [21, 49, 50]. The present study was explicitly designed to monitor the development of arm-hand use and skill performance in a broad range of stroke patients across the full stroke severity range who received a well-described ‘therapy-as-usual’, i.e. CARAS [18]. Next to evidence that a large majority of patients improved as to their AHF and AHSP, our study also provides evidence that, in a minority of patients, AHF and self-perceived AHSP deteriorate once they have left the active clinical rehabilitation setting. The latter especially holds for patients with an initially moderately or severely affected arm-hand function (subgroup 1 and 2).

With respect to the results achieved in all groups the following remarks have to be made. First, the majority of the patients with a moderately impaired arm-hand (in contrast to persons with a severely or mild impaired arm-hand) received a second six weeks period of training. This particular group of patients do have the possibility to use their affected hand again in daily performance. However, due to their moderately impaired arm–hand they need a second 6-week during period of training. Second, patients in program 1 spend 4.5 hours per week on training, while patients in program 2 and 3 spend six hours per week of training. When neurophysiological recovery is absent, patients may be left with a non-functional arm-hand, which cannot be used in daily activities. Therefore, from a clinical point of view it is not useful to train patients in program 1 (severely affected) according the same practice conditions and as intensively as patients in program 2 and 3 (i.e. moderately and mildly affected patients).

In contemporary clinical trials investigating effects of new therapy approaches patients with a moderately to severely affected hand are very often excluded. It is our firm opinion that these patient sub-groups deserve more scientific research attention regarding the exploration of the possibilities in sensorimotor training methods, especially at an early stage post-stoke.

The frame of reference of patients regarding the outcomes at the level of self-perceived AHSP may be influenced because they may reflect on their arm-hand abilities as how they were previously before the stroke occurred. The use of proxy-measurement has been considered. However, proxy-measurement might lead to other difficulties. First, regarding perceived performance it is difficult to avoid unreliable outcomes from relatives who also are familiar with the patients’ former AHSP level. Second, the measures as used in this study are not designed to be used as proxy-measurement instruments.

CARAS has *not* been proven to be superior to other arm-hand therapy approaches as described by e.g. Winstein [9], Combs [10], Arya [11], Wallace [12], McDonnell [13], Platz [14], Morris [15], and Harris [16]. As Pollock et al., [51] already highlighted, clinical decision making procedures and the clinical application of arm-hand interventions have to be tailored to the patient’s individual needs. CARAS is a clearly defined ‘therapy-as-usual’, which provides practical solutions based on the presence of dexterity and corresponding functional possibilities which facilitates a better focus and tailored therapy delivery. It provides instructions how to empower patients by using principles of self-efficacy, and allows for the systematic incorporation of (new) technology and new evidence-based training elements swiftly [18], specifically adapted to the severity of the arm-hand impairment.

## Conclusion

The present study has yielded a comprehensive longitudinal database on the development of AHF and AHSP in a broad range of stroke patients suffering from arm-hand impairments, who received well-documented ‘therapy-as-usual’, which may be used in future research as a reference database to contrast newly developed training interventions.

## Supporting information

S1 Datafile Supporting data file (SPSS format). (SAV)
